# An Innovative Use of Cortoss Bone Cement to Stabilize a Nonunion after Interbody Fusion

**DOI:** 10.7759/cureus.986

**Published:** 2017-01-20

**Authors:** Michelle Granville, Robert E Jacobson

**Affiliations:** 1 Miami Neurosurgical Center, University of Miami Hospital; 2 Coral Gables Surgery Center

**Keywords:** failed fusion, failed back syndrome, minimally invasive

## Abstract

A 65-year-old male originally had surgery for spondylolisthesis at L5-S1 in 2008 and then went on to have an L4-5 transforaminal lumbar interbody fusion (TLIF) with pedicle screw fixation from L4 to S1 and interbody graft in 2010. Despite having two surgical procedures, he continued with intractable back pain and was told he had a failed lumbar fusion. When he was evaluated with a computerized tomography (CT) scan from April 2015, it demonstrated an erosive nonunion of the L4-5 interbody fusion without incorporation of the polyetheretherketone (PEEK) cage. In an attempt to perform a minimally invasive stabilization of the L4-5 nonunion, he underwent a percutaneous lateral foraminal approach with an injection of Cortoss® cement (Stryker®, Malvern, PA) into the L4-5 interspace and around the graft. The objective was to stabilize the nonunion, resulting in intermediate relief of pain.

## Introduction

**Figure 1:**
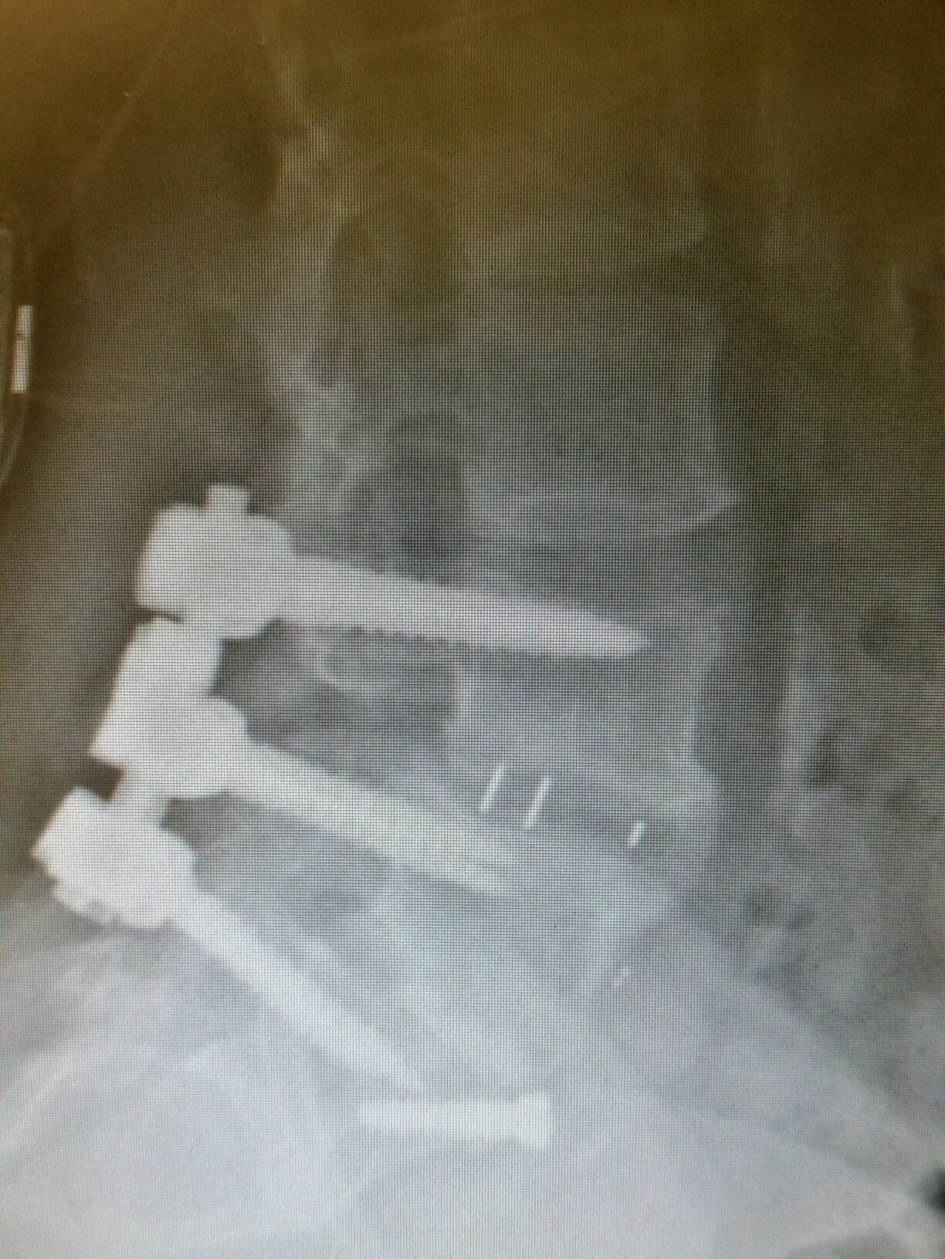
Lateral X-ray appears to show the graft in place; however, L4-5 is not fused.

**Figure 2:**
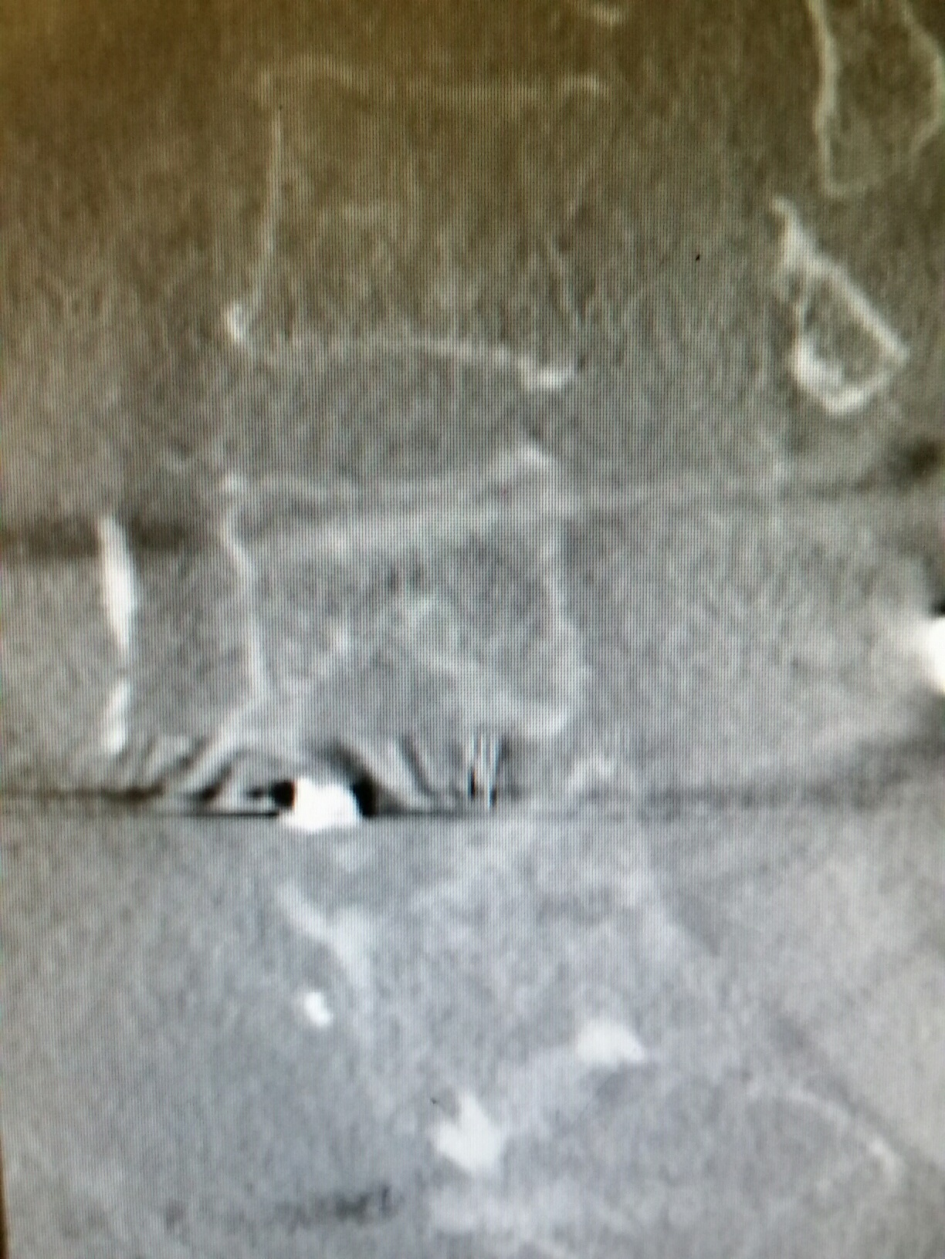
Sagittal CT reconstruction shows erosion of the endplates and rotation of the graft.

A 65-year-old male was evaluated six months after refusing surgery at another clinic for increased severe and persistent deep low lumbar pain after multiple previous lumbar surgeries. The pain actually worsened after an L4-5 interbody graft and pedicle screw fixation from L4 to S1. He was evaluated with plain X-rays and CT scan, including a fine-cut CT, which demonstrated nonunion and actual endplate subsidence and erosion both inferiorly at L4 and superiorly at L5, as shown in Figures 1-2. In order to ensure the patient did not have osteomyelitis or an underlying infection, a sedimentation rate, white blood cell (WBC) count, and bone scan were ordered and found to be within normal limits.

After careful review of all the findings, it was felt that a repeat laminectomy with an attempt to remove or replace the interbody graft posteriorly would be difficult, so the patient was offered an extreme lateral lumbar interbody fusion (XLIF) to approach the L4-5 space laterally, allowing both debridement and grafting. However, the patient refused this procedure. It was subsequently suggested that it might be possible to enter the disc space endoscopically, curette the area, and then place Cortoss®, a bioactive bone cement (Stryker®, Malvern, PA), around the graft to support the disc space. He was explained that if we could not debride and remove fibrous scar around the graft, it would be less likely the cement would be helpful. He agreed to undergo minimally invasive stabilization with Cortoss, understanding that he may have to endure another surgical procedure in the future. Informed patient consent was obtained for treatment.

## Technical report

**Figure 3:**
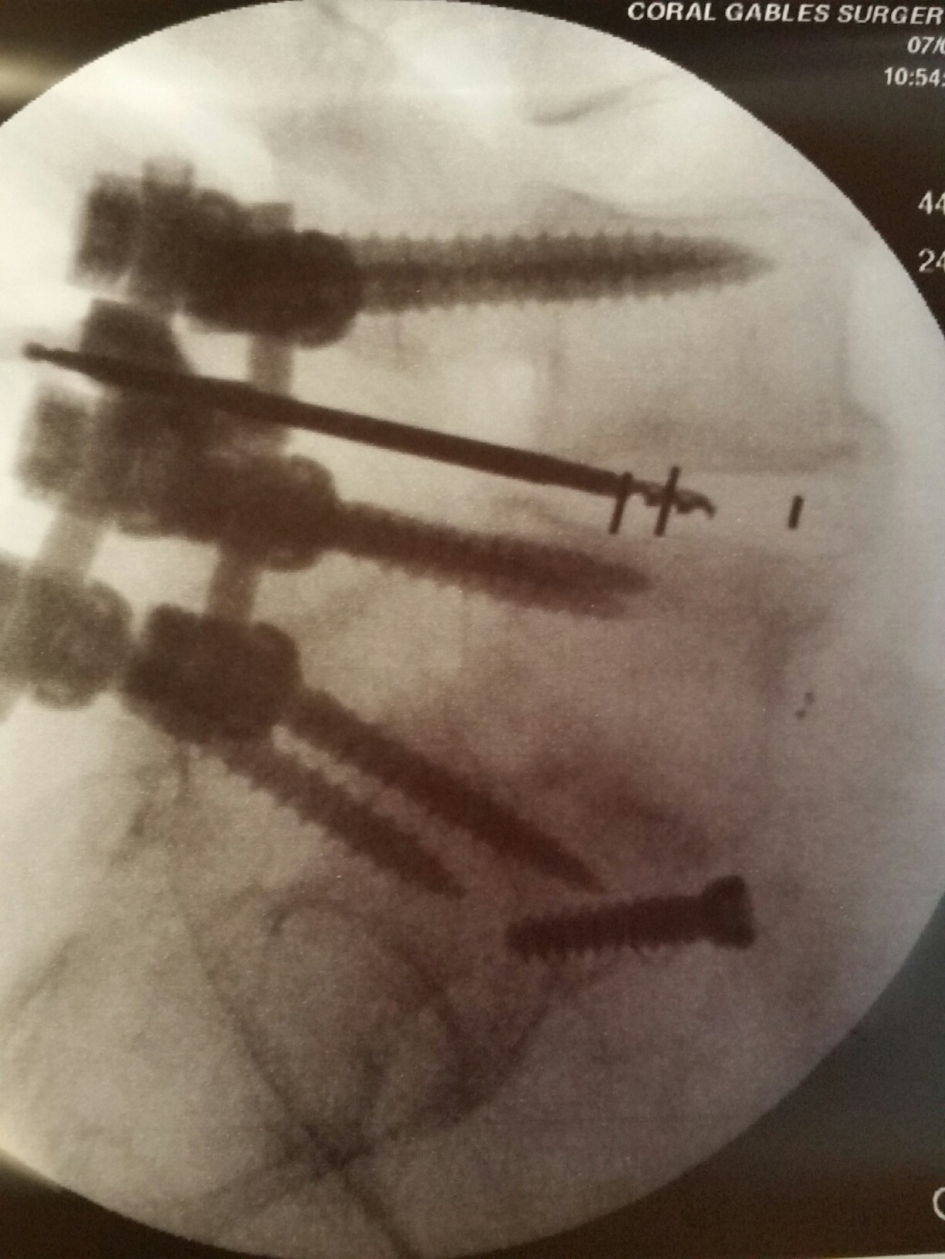
Intraoperative photograph of bone drill, curette, and pituitary ronguer debriding the L4-5 interspace.

**Figure 4:**
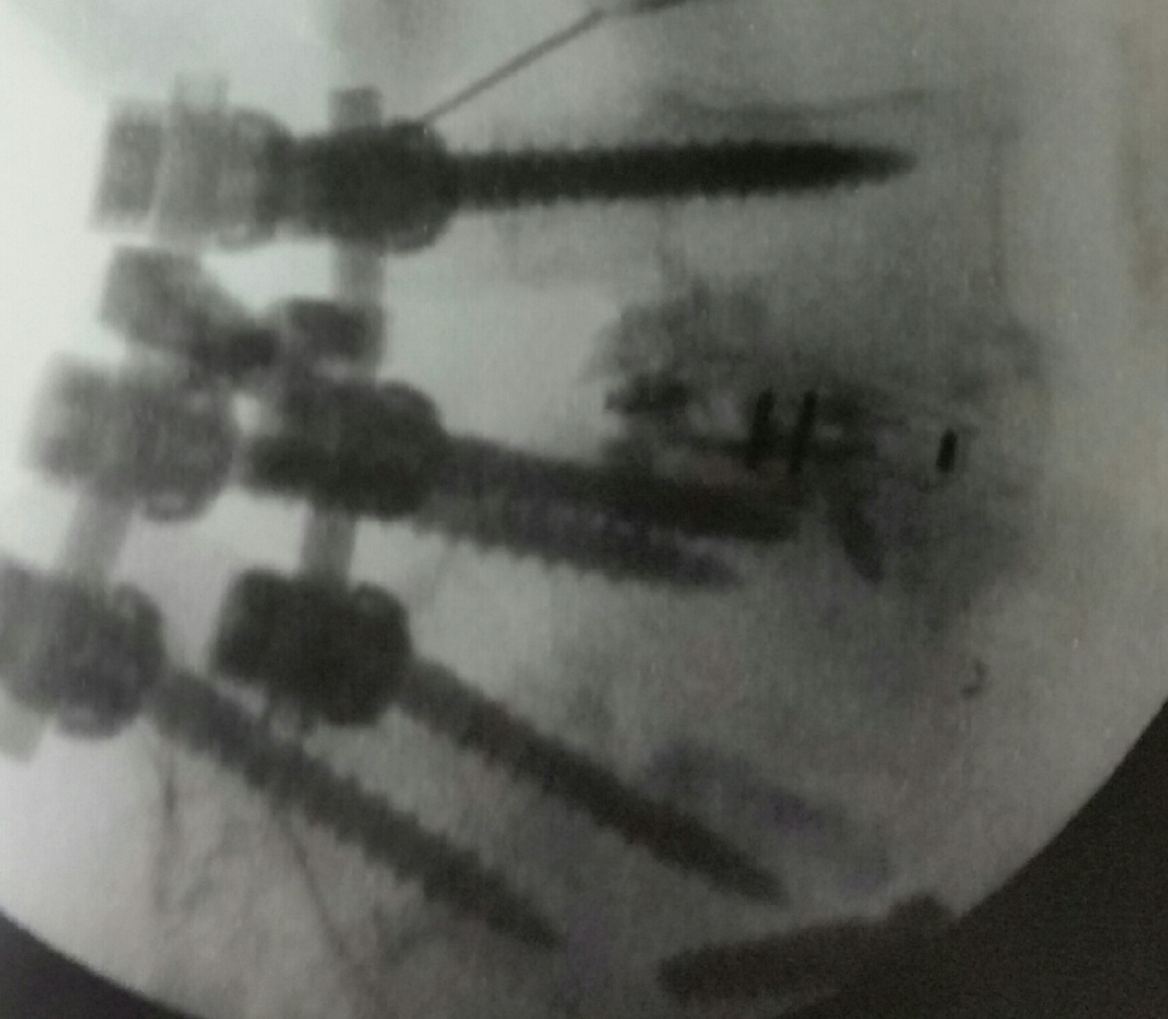
Post-procedure shows cement fill at L4-5.

The patient was taken to the procedure room and placed in a left lateral decubitus position with a roll underneath his left flank and his knees bent. He was then given monitored anesthesia care (MAC) sedation, the skin was prepped and draped, and the L4-5 region was identified with fluoroscopy. Local anesthesia with 1% lidocaine and 0.5% Marcaine was then administered to the area. Using a 20 gauge needle, anesthesia was also placed down to the foraminal margin of the disc, just under the mid-pedicle line. A guide wire was passed, and a 4 mm Stryker vertebroplasty cannula was passed along the guide wire and impacted into the posterolateral margin of the L4-5 disc space. The position was confirmed on AP and lateral fluoroscopy. A trocar was then impacted into the disc space. The annulus and outer capsule of the disc were incised with a manual spiral drill, and as the drill was passed into the disc space under fluoroscopy, the graft could be felt. The adjustable vertebroplasty curette and 3.1 mm pituitary and scope curettes were passed around the graft, as well as above and below the endplate of L4 and L5. The space was irrigated with normal saline, and as the endplate was curetted, some mild bleeding occurred indicating the bone was being debrided, as shown in Figure 3. Four pipettes were then filled with 0.7 cc of Cortoss (totaling 2.8 cc of Cortoss), which were subsequently injected into the L4-5 disc space surrounding the graft and spreading both inferiorly towards the superior margin of L5 and superiorly into the inferior margin of L4. There was minor spread along the posterior longitudinal ligament as well, but no extravasation of dye was identified, as shown in Figure 4. Then the trocar was pulled out, minor bleeding was contained, and Dermabond® (Ethicon, Somerville, NJ) and Steri-Strips™ (3M, St. Paul, MN) were placed over the incision.

The entire operative time was 33 minutes. The patient tolerated the procedure well and was sent home the same day. He was seen at the office for follow-up three days later complaining of mild soreness in his lumbar region near the incision. A six-month follow-up showed the patient had a 50% reduction in his pain.

## Discussion

Failure to fuse after lumbar surgery has numerous causes. Statistics show that advanced age and habitual smoking increase the risk and delay the healing process [1-2]. The ultimate goal of spinal fusion surgery involves setting up a natural response that causes bone to grow between two vertebras, hence, stopping motion at a given segment of the spine. This can be achieved by adding a bone graft or inserting a cage made of synthetic material, such as polyetheretherketone (PEEK) cages or titanium into the disc space [3]. It is documented that with proper preparation of the interspace and fusion bed, PEEK cages fuse at a high percentage rate when compared to titanium cages [3-4]. Posterior pedicle screw fixation over three levels should provide stability on both the posterior and middle column. However, if no adjacent facet or interlaminar fusion is performed, then failure of the anterior column PEEK cage could lead to instability since the disc space is extensively debrided of both the nucleus and some annulus in creating the bed for the PEEK cage [3]. It is well documented that settling of PEEK cages into the adjacent endplates is a significant problem and the possible cause of postoperative pain due to bone erosion as seen in this case [3-4].  

This is the first reported case of using Cortoss, especially percutaneously, through a minimally invasive tubular approach under local anesthesia as a means to stabilize a failed interbody bone graft. There are reports of using polymethylmethacrylate (PMMA), which has a higher viscosity when compared to Cortoss, for stabilizing vertebral body metastatic tumors and as an adjunct during open surgery to fill voids for vertebral body replacement [5-6]. Cortoss is the first clinically sustained substitute to PMMA, which has both calcium and phosphate polymers mixed with micro glass that binds to bone, giving it similar properties to human bone [6-7]. This also allows it to reach around 75% of the biomechanical strength of cortical bone within 15 minutes of injection [8-9]. Thus, this allowed for significant support so there was a reasonable chance of not only stabilizing the graft but actually enhancing the ability of the graft to fuse and incorporate the interbody PEEK cage and lead with the L4 and L5 endplates. 

## Conclusions

In the last several years, various methods have been developed to place PEEK cages and grafts in the disc space with the goal of increasing bony growth to allow adequate fusion. This case demonstrates that it is possible to stabilize a nonunion of a lumbar interbody graft using a variation of the technique for transforaminal lumbar endoscopic discectomy and combining this approach with the use of bone cement. This made it possible to offer the patient a minimally invasive short and median term solution to a complex interbody fusion nonunion failure.
